# Early phase clinical trials in pediatric oncology: Swedish pediatric oncologists’ experiences of balancing hope and expectations in life-threatening illnesses

**DOI:** 10.3389/fonc.2024.1395841

**Published:** 2024-08-16

**Authors:** Anna Schröder Håkansson, Ann-Christine Andersson, Jonas Abrahamsson, Margaretha Stenmarker

**Affiliations:** ^1^ Department of Pediatrics, Institute of Clinical Sciences, Sahlgrenska Academy, University of Gothenburg, Gothenburg, Sweden; ^2^ Department of Pediatrics, Queen Silvia Children’s Hospital, Gothenburg, Sweden; ^3^ Jönköping Academy, Jönköping University, Jönköping, Sweden; ^4^ Department of Care Science, Faculty of Health and Society, Malmö University, Malmö, Sweden; ^5^ Department of Biomedical and Clinical Sciences, Faculty of Medicine and Health Sciences, Linköping University, Linköping, Östergötland, Sweden; ^6^ Department of Pediatrics, Ryhov Hospital Jönköping, Jönköping, Sweden

**Keywords:** pediatric oncology, physicians, early phase clinical trials, pediatric palliative care, shared decision, children, pediatric oncologist

## Abstract

**Aim:**

To study Swedish pediatric oncologists’ practical and emotional experiences of referring, including and/or treating children in early-phase clinical trials.

**Methods:**

A nationwide study was conducted using a mixed-method approach. Structured interviews based on a study-specific questionnaire and participants’ personal reflections were utilized. Survey responses were analyzed using descriptive statistics, while participants’ comments were analyzed using thematic analysis. All interviews were recorded and transcribed verbatim.

**Results:**

In total, 29 physicians with 4 to 32 years of experience in pediatric oncology participated, with 19 (66%) having > 10 years of experience. Three themes appeared: 1) Optimization-based approach focused on finding the most suitable treatment and care for every child with a refractory/relapsed cancer eligible for an early-phase clinical trial; 2) Team-based approach aimed at establishing local and national consensus in decision-making for treatment options, including early-phase clinical trials and palliative care; 3) Family-based approach in which the physicians provided families with actionable information, listened to their desires, and endeavored to maintain hope in challenging circumstances. Several participants (40% with ≤ 10 years of experience and 58% with > 10 years of experience) viewed the early-phase clinical trial as a potential “chance of cure”. A majority (80%) of physicians with ≤ 10 years of experience, reported that they often or always felt personally and emotionally affected by communication regarding early-phase clinical trials. Delivering difficult news in cases of uncertain prognosis was identified as the major challenge. None of the study participants felt adequately prepared in terms of sufficient knowledge and experience regarding early-phase clinical trials. The physicians expressed a need for guidance and training in communication to address these challenges.

**Conclusions:**

Working with early-phase clinical trials highlight a field where physicians cannot solely rely on their expertise or past experiences, and where they are likely to be deeply emotionally involved. Physicians who care for children eligible for such studies require targeted educational initiatives and supervision.

## 1 Introduction

Around 85–90% of childhood cancers are treated according to standard protocols or in prospective clinical trials (1, 2). Despite the immense improvement in survival rates in childhood cancers, a number of patients will have a refractory disease or relapse after first-line treatment (1). For some patients with refractory disease or relapse, treatment according to evidence-based treatment protocols can be offered. However, for many patients, no established effective treatment exists, and the choice of therapy is often between conventional second-line treatments of uncertain efficacy or various novel and experimental therapies (3, 4). Ideally, these novel therapies should be given in early-phase clinical trials (EPCTs) to facilitate their development (5), but open trials are not available for all patients. Potential drugs that only have been studied in adult participants are often given in compassionate use programs for children (6). Moreover, these experimental therapies are seldom curative and the chance of cure for the individual child is low (1). At the same time, in accordance with international standards for pediatric palliative care, it is important to ensure that each child receives optimal palliative care (7).

In Sweden, when a child is diagnosed with cancer, oncological treatment is initiated at a pediatric hematology and oncology center, located at one of six university hospitals. Swedish pediatric oncologists (POs) have close national collaboration regarding all aspects of cancer therapy, related research, as well as clinical practice. Most EPCTs are conducted at the two largest university hospitals, which are members of the Consortium for Innovative Therapies for Children with Cancer (ITCC). A common national platform among Swedish POs is a weekly online meeting where difficult cases and treatment options are discussed. In addition, some difficult cases including novel therapies, potential EPCTs, and treatment given abroad are discussed at the weekly Nordic online meeting, so called NOPHO match. In this meeting different Nordic pediatric oncology centers are represented. Therefore, the Swedish pediatric oncology healthcare community could be defined as a research-integrated environment (8). POs inform and enroll patients on a regular basis in randomized late phase studies (phases 3 and 4) as part of first-line treatment. In the informed consent process, healthcare professionals are expected to provide parents and children with adequate information to enable understanding and independence in terms of autonomous decision-making (9). In line with the literature (10, 11), our previous study (8) showed that the most difficult ethical challenges for POs are related to EPCTs. Some POs consider these trials as realistic treatment options, while others do not think they provide any direct benefit to the affected child. At the same time, Swedish POs are medically responsible for all treatment decisions through the entire course of the illness, regardless of the child’s condition, that is pediatric palliative care is integrated into pediatric oncology (12, 13).

Medical development is rapidly moving toward a greater number of novel therapies. In the last decade, an increasing number of EPCTs have been available within Swedish pediatric oncology. Knowledge regarding how this development affects the working situation of Swedish POs and their experiences is lacking, but is essential, to meet the needs and demands of the future. The overall aim of this study was to survey Swedish POS regarding their practical and emotional experiences of referring, enrolling and/or treating children in EPCTs. The specific aim was to elucidate how these experiences were shaped by the level of expertise of POs within the field.

## 2 Materials and methods

### 2.1 Design

This study was performed using mixed methods research design (14, 15) with structured interviews based on a study-specific questionnaire (16). Both quantitative data from the questionnaire and qualitative data based on the POs’ oral comments were analyzed (17–19). The study was approved by the Swedish Ethical Review Authority (Dnr 2020–02386).

### 2.2 Questionnaire

A questionnaire to be use in a structured interview was developed based on our previous study (8) and appropriate literature sources. To ensure readability, clarity, and content validity, the questionnaire was reviewed by eight skilled physicians working with adult and pediatric cancer patients, including a representative of the National Council of New Therapies in Sweden. These physicians gave suggestions for further revisions. The final questionnaire comprised 22 modules, with a total of 112 items, and demographic data. Each module provided an additional free text option. Each module concluded different items related to the informed consent process from the perspective of POs practical and emotional aspects related to EPCTs.

### 2.3 Sample and participants

A purposive sampling technique was applied and included all identified possible participants, i.e., a total population. The inclusion criteria were physicians working at a pediatric hematology and oncology center at one of the six university hospitals in Sweden with experience of being part of referring, enrolling and/or treating children in EPCTs. At the time of the study (2021), there were approximately 90 physicians employed, part or full time, at the six centers. The physicians worked within different oncological subspecialties and some of the physicians were more dedicated to benign hematology diseases than oncology diseases. In the following text, each participant is referred to as a pediatric oncologist, PO.

### 2.4 Data collection

The structured interviews were conducted during 2021. All 90 POs were approached by one of the authors via an e-mail. Each PO was asked to answer the question of whether they had referred, enrolled and/or treated patients in an EPCT. All POs who answered this question with “yes” were included in the study. The POs provided consent to participate by replying via e-mail and confirmed the inclusion criteria. The structured interviews were performed by the first and the second author and mainly conducted via the digital video meeting Zoom™. One interview was performed over the telephone according to the PO’s wishes. The POs had received the questionnaire before the interview. The collection of the quantitative data and the qualitative data occurred simultaneously. All questions were read by the interviewer from the standardized questionnaire and the POs could follow the text either on shared screen or on paper. After each module, the interviewer wrote a summary of the comments which the participant could momently validate. The conversation revolved around various questions, allowing for explanations and additional comments. At times, POs revisited earlier questions to make revisions or provide further insights. The interviews were recorded after obtaining consent from the participants. The recorded audio files were transcribed verbatim by the first author.

### 2.5 Data analysis

The quantitative data were analyzed in IBM SPSS^®^ version 25.0. Descriptive statistics with frequencies, median and mean values, standard deviations (SD) and ranges were used. The study population with POs was divided into two groups. One group had experience in pediatric oncology equivalent to 10 years or less (≤ 10 years), and the second group had more than10 years (> 10 years) of experience (**Table 1**).The transcribed interviews corresponding to each of the modules in the questionnaire, including free comments and handwritten notes, were analyzed by reflexive thematic analysis, inspired by Braun and Clarke (18, 19). In accordance with Robinson (2022) (17), it is possible to make thematic analysis on brief texts such as open ended questions. The first author conducted the initial extraction and clustering of data. Thereafter, all authors were involved in the analysis. First, all extracts were entered into a common document and read through several times. The main units were identified and clustered into different subthemes, which were reviewed, refined, and compared with the full transcription. Further, the identified subthemes were clustered into themes. The themes and the descriptive statistics were discussed and elaborated on by all authors, and reflexive discussions continued until a consensus was reached. Thereafter, quotes that reflected the different themes were identified, translated from Swedish to English, and checked by a qualified language reviewer (**Table 2**).

**Table 1:** The questionnaire with the answers divided in participants with Experiences < 10 years and participants with Experiences > 10 years.

The study-specific questionnaire (Pediatric oncologists n=29)≤10 years of experience (n=10)							>10 years of experience (n=19)				
	n (%)	n (%)	n (%)	n (%)		n (%)	n (%)	n (%)	n (%)	n (%)	n (%)
Module 1. Do you have experience with early-phase clinical trials?	Never	Sometimes	Often	Always		Do not know	Never	Sometimes	Often	Always	Do not know
1a. Have been present when a colleague has informed a family?	3 (30)	4 (40)	3 (30)		0	0	4 (21.1)	12 (63.2)	3 (15.8)	0	0
1b. Have been responsible for the information?	2 (20)	5 (50)	3 (30)		0	0	3 (15.6)	10 (52.6)	5 (26.3)	1 (5.3)	0
1c. Have you treated/cared for a child/adolescent in an early-phase clinical trial?	1 (10)	5 (50)	4 (40)		0	0	1 (5.3)	14 (73.7)	3 (15.8)	1 (5.3)	0
1d. Have you been an investigator in an early-phase clinical trial?	6 (60)	3 (30)	1 (10)		0	0	11 (57.9)	5 (26.3)	2 (10.5)	1 (5.3)	0
1e. Have you referred a patient to another hospital because of an early-phase clinical trial?	7 (70)	3 (30)	0		0	0	4 (21.1)	15 (78.9)	0	0	0
1f. Have you referred a patient abroad because of an early-phase clinical trial?	6 (60)	4 (40)	0		0	0	5 (26,3)	13 (68,4)	1 (5.3)	0	0
1g. Do you have/had a patient who has left for an alternative clinic?	1 (10)	9 (90)	0		0	0	6 (31.6)	12 (63.2)	1 (5.3)	0	0
1h. Have you treated a patient who has been treated at an alternative clinic?	3 (30)	7 (70)	0		0	0	5 (26.3)	13 (68.4)	1 (5.3)	0	0
Module 2. How do you find out about a suitable/appropriate early-phase clinical trial for your patient?	Never	Sometimes	Often		Always	Do not know	Never	Sometimes	Often	Always	Do not know
2a. Via colleagues in my department.	0	0	8 (80)		2 (20)	0	3 (15.8)	9 (47.4)	5 (23.3)	2 (10.5)	0
2b. Via personal contact with colleagues in the country.	2 (20)	6 (60)	2 (20)		2 (20)	0	1 (5.3)	10 (52.6)	8 (42.1)	0	0
2c. Via the formal meeting of colleagues in Sweden.	1 (10)	3 (30)	6 (60)		0	0	1 (5.3)	10 (52.6)	6 (31.6)	2 (10.5)	0
2d. Via the formal meeting of colleagues in the Nordic countries.	9 (90)	1 (10)	0		0	0	9 (47.4)	6 (31.6)	4 (21.1)	0	0
2e. Via the trial “INFORM”.	1 (10)	8 (80)	1 (10)		0	0	1 (5.3)	11 (57.9)	7 (36.8)	0	0
2f. Via personal contact with international colleagues.	5 (50)	4 (40)	1 (10)		0	0	3 (15.8)	11 (63.2)	3 (15.8)	1 (5.3)	0
Module 3. Do you personally/your colleagues inform what the trial implies in terms of?	Never	Sometimes	Often		Always	Do not know	Never	Sometimes	Often	Always	Do not know
3a. Amount of time spent at the hospital.	0	0	4 (40)		6 (60)	0	2 (10.5)	1 (5.3)	1 (5.3)	14 (73.7)	0
3b. Amount of time spent at another hospital.	1 (40)	0	4 (40)		5 (50)	0	2 (10.5)	2 (10.5)	1 (5.3)	13 (68.4)	1 (5.3)
3c. Extra trips to the hospital.	0	1 (10)	3 (30)		6 (60)	0	1 (5.3)	1 (5.3)	1 (5.3)	14 (73.7)	2 (10.5)
3d. Expected side effects.	0	1 (10)	1 (10)		8 (80)	0	1 (5.3)	1 (5.3)	1 (5.3)	15 (78.9)	1 (5.3)
3e. Different/alternative treatment option.	1 (10)	1 (10)	7 (70)		1 (10)	0	2 (10.5)	1 (5.3)	1 (5.3)	15 (78.9)	0
Module 4. Have you/your colleagues ever chosen not to inform about an early-phase clinical trial because …	Never	Sometimes	Often		Always	Do not know	Never	Sometimes	Often	Always	Do not know
4a. Someone/some in the family has/have language difficulties (Swedish as a second language)?	7 (70)	2 (20)	0		0	1 (10)	12 (63.2)	4 (21.1)	1 (5.3)	2 (10.5)	0
4b. Someone/some in the family has/have mental illness?	10 (100)	0	0		0	0	14 (73.7)	4 (21.1)	0	0	1 (5.3)
4c. The family has expressed that they want to hand over the decision to you as a doctor.	10 (100)	0	0		0	0	15 (78.9)	2 (10.5)	0	0	2 (10.5)
4d. Someone/some in the family has/have difficulties understanding the design of the study (e.g., illiteracy)?	7 (70)	2 (20)	0		0	1 (10)	11 (57.9)	6 (31.6)	0	0	2 (10.5)
4e. The child/adolescent does not live with the parents?	9 (90)	1 (10)	0		0	0	16 (84.2)	1 (5.3)	0	0	2 (10.5)
Module 5. When you inform about an early-phase clinical trial how do you do it?	Never	Sometimes	Often		Always	Do not know	Never	Sometimes	Often	Always	Do not know
5a. With the whole family gathered (child and parents together).	0	0	5 (50)		5 (50)	0	1 (5.3)	1 (5.3)	8 (42.1)	8 (42.1)	1 (5.3)
5b. Alone with the parents.	3 (30)	3 (30)	3 (30)		1 (10)	0	5 (26.3)	8 (42.1)	3 (15.8)	2 (10.5)	1 (5.3)
5c. Alone with the teen 15-18 years of age.	3 (30)	2 (20)	3 (30)		0	2 (20)	6 (31.6)	5 (26.3)	6 (31.6)	1 (5.3)	1 (5.3)
5d. Alone with the teen 13- <15 years of age.	4 (40)	3 (30)	0		0	3 (30)	11 (5.,9)	3 (15.8)	3 (15.8)	1 (5.3)	1 (5.3)
5e. Alone with child <13 years of age.	8 (80)	1 (10)	1 (10)		0	0	12 (63.2)	6 (31.6)	0	0	1 (5.3)
Module 6. In your opinion, what do you think is a “perfect” information setting regarding an early-phase clinical trial?	Never	Sometimes	Often		Always	Do not know	Never	Sometimes	Often	Always	Do not know
6a. With the whole family gathered (child and parents together).	0	0	1 (10)		9 (90)	0	1 (5.3)	1 (5.3)	5 (26.3)	12 (63.2)	0
6b. Alone with the parents.	1 (10)	3 (30)	1 (10)		5 (50)	0	3 (15.8)	9 (47.4)	3 (15.8)	4 (21.1)	0
6c. Alone with the teen 15-18 years of age.	3 (30)	1 (10)	2 (20)		4 (40)	0	3 (15.8)	3 (15.8)	5 (26.3)	8 (42.1)	0
6d. Alone with the teen 13- <15 years of age.	4 (40)	2 (20)	3 (30)		1 (10)	0	3 (15.8)	8 (42.1)	4 (21.1)	4 (21.1)	0
6e. Alone with child <13 years of age.	5 (50)	1 (10)	3 (30)		1 (10)	0	7 (36.8)	8 (42.1)	2 (10.5)	2 (10.5)	0
Module 7. Who is the person who informs the patient/patient’s family about an early-phase clinical trial at your clinic?	Never	Sometimes	Often		Always	Do not know	Never	Sometimes	Often	Always	Do not know
7a. You as the treating physician.	1 (10)	1 (10)	3 (30)		4 (40)	1 (10)	2 (10.5)	1 (5.3)	10 (52.6)	6 (31.6)	0
7b. Another physician at your clinic.	1 (10)	5 (50)	3 (30)		0	1 (10)	5 (26.3)	11 (57.9)	2 (10.5)	1 (5.3)	0
7c. A dedicated study physician.	3 (30)	2 (20)	1 (10)		4 (40)	0	1 (5.3)	7 (36.8)	7 (36.8)	4 (21.1)	0
Module 8. When a family has chosen not to enrol, have you experienced that…	Never	Sometimes	Often		Always	Do not know	Never	Sometimes	Often	Always	Do not know
8a. The parents wanted to enrol but not the child/adolescent?	6 (60)	2 (20)	1 (10)		0	2 (20)	11 (57.9)	5 (26.3)	0	0	3 (15.8)
8b. One of the parents did not want to enrol.	5 (50)	2 (20)	1 (10)		0	2 (20)	12 (63.2)	4 (21.1)	0	0	3 (16.7)
8c. The child/adolescent wanted to enrol but not the parents?	7 (70)	0	1 (10)		0	2 (20)	14 (73.7)	2 (10.5)	0	0	3 (15.8)
Module 9. Have you enrolled a child/adolescent but sensed/felt that…	Never	Sometimes	Often		Always	Do not know	Never	Sometimes	Often	Always	Do not know
9a. The teen (15-18) wanted but not the parents?	9 (90)	1 (10)	0		0	0	15 (78.9)	1 (5.3)	0	0	3 (15.8)
9b. The teen (13-<15) wanted but not the parents?	9 (90)	1 (10)	0		0	0	15 (78.9)	1 (5.3)	0	0	3 (15.8)
9c. The child (<13) wanted but not the parents?	10 (100)	0	0		0	0	15 (78.9)	1 (5.3)	0	0	3 (15.8)
9d. It was the parents’ will and not the teen’s (15-18)?	7 (70)	2 (20)	0		0	1 (10)	12 (63.2)	2 (10.5)	1 (5.3)	0	4 (21.1)
9e. It was the parents’ will and not the teen’s (13-<15)?	6 (60)	3 (30)	0		0	1 (10)	12 (63.2)	3 (15.8)	0	0	4 (21.1)
9f. It was the parents’ will and not the child’s (<13)?	5 (50)	3 (30)	1 (10)		0	1 (10)	12 (63.2)	3 (15.8)	0	1 (5.3)	3 (15.8)
Module 10. Have you ever chosen to not inform because you did not…	Never	Sometimes	Often		Always	Do not know	Never	Sometimes	Often	Always	Do not know
10a. Did not have enough knowledge about the specific early-phase clinical trial?	7 (70)	2 (20)	1 (10)		0	0	15 (78.9)	1 (5.3)	2 (10.5)	0	1 (5.3)
10b. Did not understand the aim of the study?	9 (90)	1 (10)	0		0	0	17 (89.5)	1 (5.3)	0	0	1 (5.3)
10c. Where short on time?	9 (90)	1 (10)	0		0	0	17 (89.5)	1 (5.3)	0	0	1 (5.3)
10d. Have been advised by a colleague to not bring up the early-phase clinical trial with family?	7 (70)	2 (20)	0		0	1 (10)	15 (78.9)	3 (15.8)	0	0	1 (5.3)
Module 11. Have you yourself questioned the usefulness of a specific early-phase clinical trial because you thought/had…	Never	Sometimes	Often		Always	Do not know	Never	Sometimes	Often	Always	Do not know
11a. The study had a bad scientific question or design?	4 (40)	4 (40)	1 (10)		0	1 (10)	9 (47.4)	9 (47.4)	0	0	1 (5.3)
11b. The study would have unjustified risk or cause discomfort to the child/adolescent?	2 (20)	7 (70)	1 (10)		0	0	6 (31.6)	9 (47.4)	4 (21.1)	0	0
11c. Another opinion about the usefulness of the trial than your colleagues?	8 (80)	1 (10)	1 (10)		0	0	6 (31.6)	9 (47.4)	4 (21.1)	0	0
Module 12. Have you ever felt that your opinion of the trial has been the reason…	Never	Sometimes	Often		Always	Do not know	Never	Sometimes	Often	Always	Do not know
12a. That the family has consent, a joint decision?	1 (10)	4 (40)	4 (40)		0	1 (10)	9 (47.4)	5 (26.3)	1 (5.3)	1 (5.,3)	3 (15.8)
12b. That the parents want to consent/enrol?	1 (10)	5 (50)	3 (30)		0	1 (10)	9 (47.4)	6 (31.6)	1 (5.3)	0	3 (15.8)
12c. Child/adolescent wants to consent/enrol?	3 (30)	4 (40)	2 (20)		0	1 (10)	9 (47.4)	4 (21.1)	1 (5.3)	0	5 (26.3)
12d. The family did not want to consent/enrol?	4 (40)	3 (30)	2 (20)		0	01 (10)	8 (42.1)	6 (31.6)	0	0	5 (26.3)
12e. Parents did not want to consent/enrol?	4 (40)	3 (30)	2 (20)		0	1 (10)	8 (42.1)	6 (31.6)	0	0	5 (26.3)
Module 13. When you have informed about enrolling in an early-phase clinical trial would you say that …	Never	Sometimes	Often		Always	Do not know	Never	Sometimes	Often	Always	Do not know
13a. The parents made an independent decision.	0	0	6 (60)		4 (40)	0	0	0	6 (31.6)	12 (63.2)	1 (5.3)
13b. The child/adolescent made an independent decision.	1 (10)	2 (20)	6 (60)		1 (10)	0	1 (5.3)	6 (31.6)	5 (26.3)	3 (15.8)	4 (21.1)
13c. The parents did not make an independent decision.	1 (10)	2 (20)	5 (50)		1 (10)	1 (10)	4 (21.1)	2 (10.5)	1 (5.3)	9 (47.4)	3 (15.8)
13d. The child/adolescent did not make an independent decision.	3 (30)	1 (10)	4 (40)		1 (10)	1 (10)	6 (31.6)	2 (10.5)	2 (10.5)	4 (21.1)	5 (26.3)
Module 14. Would you say that participation in an early-phase clinical trial generally …	Never	Sometimes	Often		Always	Do not know	Never	Sometimes	Often	Always	Do not know
14a. Is by the will of the child/adolescent?	0	2 (20)	7 (70)		1 (10)	0	0	6 (31.6)	8 (42.1)	4 (21.1)	1 (5.3)
14b. Are by the will of the parents?	0	0	5 (50)		5 (50)	0	1 (5.3)	0	11 (57.9)	7 (36.8)	0
14c. Are by the will of the relatives?	5 (50)	2 (20)	0		0	3 (30)	10 (52.6)	3 (15.8)	0	0	6 (31.6)
14d. Is against the will of the child/adolescent?	8 (80)	1 (10)	0		0	1 (10)	14 (73.7)	4 (21.1)	0	0	1 (5.3)
Module 15. What do you believe the parents think of an early-phase clinical trial?	Never	Sometimes	Often		Always	Do not know	Never	Sometimes	Often	Always	Do not know
15a. The drug prolongs the child’s life.	0	1 (10)	5 (50)		4 (40)	0	0	1 (5.3)	12 (63.2)	6 (31.6)	0
15b. The drug gives the child a chance of cure.	1 (10)	2 (20)	5 (50)		2 (20)	0	2 (10.5)	4 (21.1)	10 (52.6)	3 (15.8)	0
15c. The study gives hope to the child/adolescent.	0	1 (10)	4 (40)		4 (40)	1 (10)	0	3 (15.8)	9 (47.4)	7 (36.8)	0
15d. The study is a way of developing new treatments.	1 (10)	2 (20)	3 (30)		3 (30)	1 (10)	1 (5.3)	7 (36.8)	6 (31.6)	4 (21.1)	1 (5.3)
15e. That their child’s/teen’s involvement will help others (future patients).	2 (20)	1 (10)	2 (20)		5 (50)	0	0	8 (42.1)	6 (31.6)	5 (26.3)	0
Module 16. What do you believe the child/adolescent thinks of an early-phase clinical trial?	Never	Sometimes	Often		Always	Do not know	Never	Sometimes	Often	Always	Do not know
16a. The drug prolongs the child’s life.	0	0	6 (60)		3 (30)	1 (10)	1 (5.3)	5 (26.3)	10 (52.6)	3 (15.3)	0
16b. The drug gives the child a chance of cure.	1 (10)	2 (20)	6 (60)		1 (10)	0	3 (15.3)	4 (21.1)	7 (36.8)	5 (26.3)	0
16c. The study gives hope to the child/adolescent.	0	0	5 (50)		4 (40)	1 (10)	1 (5.3)	3 (15.8)	9 (47.4)	6 (31.6)	0
16d. The study is a way of developing new treatments.	2 (20)	2 (20)	5 (50)		0	1 (10)	1 (5.3)	9 (47.4)	7 (36.8)	1 (5.3)	1 (5.3)
16e. That their child’s/teen’s involvement will help others (future patients).	2 (20)	3 (30)	4 (40)		0	1 (10)	1 (5.3)	10 (52.6)	5 (26.2)	2 (10.5)	1 (5.3)
Module 17. Do you say that the treatment could prolong life when you are talking with ….	Never	Sometimes	Often		Always	Do not know	Never	Sometimes	Often	Always	Do not know
17a. Parents?	4 (40)	2 (20)	2 (20)		2 (20)	0	7 (36.8)	9 (47.4)	1 (5.3)	2 (10.5)	0
17b. Older teens (15-18)?	4 (40)	1 (10)	2 (20)		3 (30)	0	7 (36.8)	9 (47.4)	1 (5.3)	2 (10.5)	0
17c. Younger teens (13-<15)?	5 (50)	2 (20)	1 (10)		2 (20)	0	7 (36.8)	8 (42.1)	1 (5.3)	2 (10.5)	1 (5.3)
17d. Children (<13)?	5 (50)	1 (10)	1 (10)		3 (30)	0	7 (36.8)	7 (36.8)	1 (5.3)	2 (10.5)	2 (10.5)
Module 18. Do you say that the treatment could lead to a cure when you are talking with …	Never	Sometimes	Often		Always	Do not know	Never	Sometimes	Often	Always	Do not know
18a. Parents?	8 (80)	0	1 (10)		0	1 (10)	15 (78.9)	2 (10.5)	0	2 (10.5)	0
8b. Older teens (15-18)?	8 (80)	0	1 (10)		0	1 (10)	15 (78.9)	2 (10.5)	0	2 (10.5)	0
18c. Younger teens (13-<15)?	8 (80)	1 (10)	0		0	0	15 (78.9)	2 (10.5)	0	2 (10.5)	0
18d. Children (<13)?	7 (70)	1 (10)	1 (10)		0	1 (10)	16 (84.2)	1 (5.3)	0	2 (10.5)	0
Module 19. Your opinion in general is that an early-phase clinical trial …	Never	Sometimes	Often		Always	Do not know	Never	Sometimes	Often	Always	Do not know
19a. Gives hope to the child/adolescent.	0	3 (30)	4 (40)		3 (30)	0	1 (5.3)	4 (21.1)	9 (47.4)	5 (26.3)	0
19b. Gives hope to the parents.	0	1 (10)	6 (60)		3 (30)	0	1 (5.3)	3 (15.8)	10 (52.6)	5 (26.3)	0
19c. Prolong life.	0	6 (60)	3 (30)		0	1 (10)	2 (10.5)	16 (84.2)	1 (5.3)	0	0
19d. Is a chance of cure.	5 (50)	4 (40)	0		0	1 (10)	8 (42.1)	10 (52.6)	1 (5.3)	0	0
19e. Is more discomfort than help for the child/adolescent.	2 (20)	7 (70)	1 (10)		0	0	3 (15.3)	12 (63.2)	4 (21.1)	0	0
19f. Can be combined with palliative care.	1 (10)	0	3 (30)		4 (40)	2 (20)	0	4 (21.1)	8 (42.1)	6 (31.6)	1 (5.3)
19g. Exclude palliative care.	6 (60)	3 (30)	0		1 (10)	0	14 (73.7)	4 (21.1)	0	0	1 (5.3)
Module 20. Has your unit been hesitant to inform about a specific early-phase clinical trial due to any of the following reasons?	Never	Sometimes	Often		Always	Do not know	Never	Sometimes	Often	Always	Do not know
20a. The trial is not useful for the child.	0	6 (60)	1 (10)		1 (10)	2 (20)	7 (36.8)	7 (36.8)	3 (15.8)	1 (5.3)	1 (5.3)
20b. The trial can be harmful to the child.	1 (10)	4 (40)	1 (10)		2 (20)	2 (20)	7 (36.8)	6 (31.6)	3 (15.8)	1 (5.3)	2 (10.5)
20c. The trial is judged to cause suffering.	1 (10)	4 (40)	2 (20)		2 (20)	1 (10)	5 (26.3)	8 (42.1)	3 (15.8)	1 (5.3)	2 (10.5)
20d. Participation in a trial will burden your unit regarding staffing.	7 (70)	2 (20)	0		0	1 (10)	13 (68.4)	5 (26.3)	1 (5.3)	0	0
20e. Participation in a trial will burden your unit (healthcare) financially (is too costly).	8 (80)	1 (10)	0		0	1 (10)	13 (68.4)	6 (31.6)	0	0	0
Module 21. Has your unit refrained from participating in a specific early-phase clinical trial due to any of the following reasons?	Never	Sometimes	Often		Always	Do not know	Never	Sometimes	Often	Always	Do not know
21a. The trial is not useful for the child.	2 (20)	4 (40)	1 (10)		0	3 (30)	4 (21.1)	10 (52.6)	2 (10.5)	0	3 (15.8)
21b. The trial can be harmful to the child.	2 (20)	3 (30)	1 (10)		0	4 (40)	7 (36.8)	7 (36.8)	1 (5.3)	0	4 (21.1)
21c. The trial is judged to cause suffering.	3 (30)	3 (30)	1 (10)		0	3 (30)	4 (21.1)	9 (47.4)	2 (10.5)	0	4 (21.1)
21d. Participation in a trial will burden your unit (healthcare) regarding staffing.	5 (50)	1 (10)	0		0	4 (40)	14 (73.7)	3 (15.8)	1 (5.3)	0	1 (5.3)
21e. Participation in a trial will burden your unit (healthcare) financially (is too costly).	5 (50)	1 (10)	0		0	4 (40)	15 (78.9)	3 (15.8)	0	0	1 (5.3)
Module 22. Early-phase clinical trials are for those children who have a relapse or refractory disease. This situation/conversation is something that many in pediatric oncology are emotionally affected by. When you think about that situation/conversation. Is there any time when you…	Never	Sometimes	Often		Always	Do not know	Never	Sometimes	Often	Always	Do not know
22a. Have discussed the good of the trial?	0	1 (10)	3 (30)		6 (60)	0	0	2 (10.5)	10 (52.6)	7 (36.8)	0
22b. Felt forced to inform?	7 (70)	3 (30)	0		0	0	14 (73.7)	3 (15.8)	2 (10.5)	0	0
22c. Felt personally affected?	1 (10)	1 (10)	3 (30)		5 (50)	0	5 (26.3)	5 (26.3)	5 (26.3)	3 (15.8)	1 (5.3)
22d. Felt personally affected that it had an impact on your information to the family?	6 (60)	3 (30)	0		0	1 (10)	13 (68.4)	5 (26.3)	0	1 (5.3)	0
22e. Wished for ethical guidance regarding early-phase clinical trials?	1 (10)	6 (60)	3 (30)		0	0	2 (10.5)	13 (68.4)	1 (5.3)	3 (15.3)	0
Module 23. If there was an education in how to inform about early phase clinical trials, what should it include?	Experiences ≤ 10 years (n=10)						Experiences > 10 years (n=19)						
	Yes n (%)	No n (%)	Maybe n (%)			Do not know n (%)	Yes n (%)	No n (%)	Maybe n (%)		Do not know n (%)
23a. Communication methodology.	8 (80)	0	2 (20)			0	16 (84,2)	0	2 (10,5)		1 (5,3)
23b. Seminaries.	9 (90)	0	1 (10)			0	11 (57,9)	2 (10,5)	5 (26,3)		1 (5,3)
23c. Discussions in groups.	9 (90)	0	1 (10)			0	14 (73,7)	0	5 (26,3)		0
23d. Ethics- debriefing/tutor about ethical implications of early phase clinical trials.	9 (90)	1 (10)	0			0	18 (94,7)	1 (5,3)	0		0
23e. Palliative care- debriefing/tutor about palliative care in combination with early phase clinical trials.	9 (90)	0	1 (10)			0	18 (94,7)	1 (5,3)	3 (15,8)		0

**Table 2:** Themes, sub-themes and quotations.

Theme	Sub-theme	Sample quotes
Optimization -based approach	*Treatment decisions*	A1: “You try to present this as an approach in palliative care, an approach to slow down illness or prolong life.” A2: There are different answers depending on the phase. In phase 1, there is no guarantee of extended life or cure, while in phase 2, it is a possibility.” A3: “… felt doubtful about it … because it involves to many unnecessary risks for the patient.” A4: “I have never experienced that they [the family] did not want to participate”.
	*Maintain hope*	A5: “I think no one would participate if it did not carry any kind of hope”. A6: “… sometimes the expectations are not realistic, you cannot inform in a way that takes away that hope.” A 7: “You may question why offer a treatment that may not prove effective, but it can be crucial for the young person to try a study drug. As a healthcare professional, it is important to approach hope with humility, recognizing its significance for the well-being of young individuals. While hope can foster feelings of self-worth, it is essential not to exaggerate its impact and instead remain modest about its implications.”
Team-based approach	C*onsensus in decision-making*	B1: “It is never the case that you refer (include) a patient yourself, it is a joint decision of the medical team” B2: “When you inform about phase 1 and 2 studies, it always precedes of a discussion between colleagues about the patient and the suitability of the study … not done by a single physician” B3 “We try to, from our different competences, decide what’s best to do”.
	*Experience* *and skills*	B4: “… haven’t experienced so many” “It is so few studies that I have been involved in”. B4: “How to inform about studies, I have mainly learned from colleagues” B5: “This is palliative situation; you feel emotionally affected¨. Otherwise, it would be strange.” B6: “It is different depending on the study and the situation”
Family-based approach	*Applied information*	C1: “You must adapt the information to what the patient wants, the family’s wishes” C2: “It depends on what the teenager wants if I talk to them individually” C3: “It depends on the child’s age”
	*Family’s desire*	C4: “I cannot think of any occasion where someone in the family did not want to. If they have refrained, they have been united.” C5: “… but I wonder how they [the family] understand what it means that the treatment is a phase 1 or phase 2 study” C6: “I could say it prolongs life, but never that it is a cure”

## 3 Results

In total, 31 POs fulfilled the inclusion criteria, and 29 POs (16 females, 13 males) were interviewed. One PO declined participation due to time constraints, while the other is one of the co-authors of the study. The majority of participating POs (n=28) were specialists in pediatric oncology. The other PO was trained as a pediatrician and had almost finished the pediatric oncology training. The characteristics of the study participants, the POs, (n=29) are presented in **Table 3**. In accordance with the literature, POs with more than 10 years of experience in pediatric oncology were defined as experienced (19, 20). The survey results are presented divided into POs with <10 years (n=10) of experience and POs with >10 years (n=19) of experience (**Table 1**). The comments and the descriptive statistics are interpreted and integrated into the results. Three themes emerged: *Optimization-based approach, Team-based approach*, and *Family-based approach*. An overview of the results is presented in **Table 2**, including representative quotes, which are referred to in the text.

**Table 3:** Description of the study population (n=29).

Characteristics	
Gender n (%)	
Female	16 (55.2)
Male	13 (44.8)
Age Years range (m)	36-63 (51,5)
Medical career n (%)	
Specialist in pediatric oncology	28 (96.4)
Resident	1 (3.6)
Experiences within pediatric oncology n (%)	
≤ 10 years	10 (34.5)
> 10 years	19 (65.5)
Range (years)	4-32
Mean (years)	15,88
Median (years)	14

The theme *Optimization-based approach* reflects how the POs tried to find the most suitable treatment and care for the individual child and family. The theme consists of the subthemes *treatment decisions* and *maintaining hope.* In most cases, the intent of treatment in the EPCT was not curative, it was symptomatic relief in line with pediatric palliative care, and the POs were eager to find the most suitable treatment. Ten percent of less experienced POs reported that they often presented an EPCT as treatment alternative that “could lead to a cure”. Ten percent of more experienced reported that they sometimes presented the EPCT in the same way. Furthermore, 10% of the more experienced POs reported that they always said that an EPCT “could lead to a cure” when informing the family (**Table 1**, module 19). On one hand, the POs tried to present an EPCT to the family as an option to prolong the child’s life and not as a cure (**Table 2**, A1). On the other hand, when talking to parents and children, some POs reported that they said that an EPCT “could lead to a cure”. An EPCT as a treatment alternative which “could be a chance of cure” was reported by 40% of the less experienced POs’ and 58% of the more experienced ones. The POs commented on their ratings, among other things, based on their perception of differences between phase 1 and phase 2 studies. (**Table 1**, module 19, item 19d; **Table 2**, A2). Most of the POs, 70% with less experience and 74% with more experience, reported that EPCT could be integrated with pediatric palliative care. However, many POs believed that EPCT exclude palliative care “sometimes” (30% of less experienced and 21% of more experienced), or “always” (10% with less experience) (**Table 1**, modules 18–19). In deciding to refer a child to an EPCT, the PO’s considered the potential of the EPCT and/or treatment (**Table 1**, modules 10–11), and how enrolment would affect the child’s situation.

The POs also emphasized that if they thought an EPCT was not beneficial or could be harmful, they did not suggest the EPCT to the family. This was apparent in the oral statements and the questionnaire answers (**Table 1**, module 20; **Table 2**, A3). The POs emphasized that it was very rare that a family did not wish to participate in an EPCT (**Table 2**, A4).

The POs wanted to preserve the family’s hope as they did not know if an EPCT could be curative. Therefore, they seldom refrained from informing the family and the child about a possible EPCT option (**Table 1**, module 4). This was true even if the POs sometimes believed that both the parents and the child hoped for a cure by participating in an EPCT (**Table 1**, modules 15–16). The POs expressed that it was crucial to support the families in not losing hope (**Table 2**, A5, A6, A7) and their opinion was that an EPCT gave hope to the whole family (**Table 1**, module 19). Offering access to an experimental therapy was a way to take an ‘active’ approach and take action in a serious, life-limiting situation. The POs felt that taking action could convey a sense of hope, although sometimes, they found this to be difficult to achieve when hope was not realistic (**Table 2**, A6, A7).

The theme *Team-based approach* highlights the importance of not making decisions regarding referral to EPCTs alone and comprises the subthemes c*onsensus in decision-making* and *experience and skills.* In general, patients with refractory disease or relapse were considered rare and their treatment involved all colleagues at the department. Senior colleagues supervised the less experienced POs in a joint discussion regarding important decisions. The team with colleagues at the local and national level, supported the individual PO in deciding which treatment would suit the child best (**Table 2**, B1). All less experienced POs discussed the decision with more experienced colleagues at their own departments. In contrast, those with more than 10 years of experience often discussed with other experienced colleagues in national and Nordic networks (**Table 1**, module 2). The POs emphasized that they were not alone in making medical decisions regarding patients who were eligible for an EPCT (**Table 2**, B2). Thus, reaching a consensus gave the PO confidence when discussing treatment options with the family.

The sub-theme *experience and skills* deals with the role of good knowledge when informing families and children about EPCTs (**Table 1**, modules 1 and 3). Working in collaboration with POs, both at the local and national level, the POs were supported emotionally and practically by more experienced colleagues (**Table 2**, B3). While several POs had senior positions within pediatric oncology, none of the POs felt that they had solid knowledge or sufficient experience including patients in EPCTs (**Table 2**, B4). Limited experience with EPCTs was a recurring issue that was brought up in the interviews. POs expressed that it was important to be well-versed with specific EPCTs (**Table 1**, modules 10–11) and wished for more education in the consent process around EPCTs (**Table 1**, module 23). In their present practice, POs often learned from each other or a more experienced colleague. Eighty percent of the less experienced POs, often or always felt personally and emotionally affected by communication regarding EPCTs. In the group of more experienced POs, they were more divided in their responses, ranging from never to always being personally and emotionally affected (**Table 1**, module 22). One experienced PO stated that personal emotional engagement could have an impact on the information communicated around EPCTs to the family (**Table 2**, B5). In addition, POs shared that the situation of the child and the family and the EPCT design could have an impact on their personal experiences (**Table 2**, B6). Regardless of their level of experience, POs considered communication training as important. Seminars and group discussions were particularly important for 80–90% of those less experienced in pediatric oncology. The importance of tutor support was emphasized by those in both groups, especially among the more experienced POs (**Table 1**, module 23).

The theme *Family-based approach* consists of the subthemes *applied information* and *the family’s desire*. The POs described how they adjusted the information they offered to each family (**Table 1**, modules 3–4; **Table 2**, C1), and strived to meet the family’s desire regarding the treatment of the child (**Table 1**, modules 13–16). Most families were well-known to the PO since he or she had cared for the child and the family over the course of the child’s cancer journey. Information regarding treatment response or cancer recurrence and treatment options was almost always given to the parents with the child present (**Table 1**, modules 5–6). However, some POs stated that it was important to offer information to children based on child’s age and independently to older children (**Table 2**, C2-C3).

The POs perceived that most families wanted additional therapy, and few families were ready for the transition to care focused solely on symptom control. Most families wanted to “buy time” and were willing to take the risk of side effects when the child was offered enrolment in an EPCT (**Table 1**, module 12). The POs perceived that the internal family conversations commonly ended in a decision to participate in an EPCT (**Table 2**, C4). However, at the same time, POs felt that families did not always understand the primary aim of an EPCT (**Table 2**, C5), or that the child could take part in an EPCT and receive palliative care at the same time (**Table 1**, module 19). Sometimes, families thought that palliative care could be postponed, even though POs seldom said that an EPCT could offer a cure, and in the best case, prolong life (**Table 2**, C6).

## 4 Discussion

This study presents data on the perspectives of POs who are treating children with incurable cancer during life-limiting circumstances when an EPCT could be a treatment option. We identified three main themes, *Optimization-based approach*, *Team-based approach* and *Family-based approach*, related to the goal of POs to, in collaboration with colleagues, optimize the possibility of finding the best treatment for the child while maintaining a focus on the family’s needs and wishes. Regardless of their level of expertise in pediatric oncology, participating POs found introducing an EPCT to a family as one of their most difficult work-related challenges (20).

In Sweden, previous research has shown that POs take responsibility for a sick child’s medical needs from diagnosis to the end of treatment, regardless of outcome (12, 21). This responsibility reflects the fact that pediatric oncology care and palliative care are often integrated. At the time when an EPCT becomes a treatment option, our study confirms that POs face several challenges related to communication (21, 22). Pediatric oncologists have the responsibility to disclose a poor prognosis and at the same time discuss different treatment options, aiming to relieve symptoms. Furthermore, our study results show that an EPCT can be a way for physicians to express a treatment option (23, 24), as approximately half of the POs considered that an EPCT could be “a chance of cure”. This process involves a balancing act between maintaining realistic expectations and quality of life and keeping a glimmer of hope alive (24, 25). The need for balance is not only for the sake of the family but also may serve as a coping strategy for the PO to handle his or her emotional involvement. An interesting finding is that approximately one in ten of the more experienced POs reported that they always viewed EPCT as an opportunity for curative treatment. These findings need further analysis. Our interpretation is that an EPCT can symbolize taking an ‘active’ action, which evokes beliefs of an increased chance of cure and maintains hope, not only for the family but also for the PO. Thus, the action of a PO to refer a patient to an EPCT can be considered a strategic component of providing optimal care for a vulnerable patient group. However, the POs’ way of expressing themselves could lead to misunderstandings regarding the treatment’s potential to affect the disease. It is crucial to underline that informed consent is about parents and children consenting to participate in a research study to test a new drug, not that the medication will lead to a cure for the current patient (26). Transparency and clear communication help protect patient autonomy by allowing them to make decisions with complete information, and potentially prevent them from being harmed by non-beneficial participation in EPCTs.

Knowledge is crucial when introducing new treatment options, however, POs face challenges in obtaining a sufficient level of knowledge regarding EPCTs. The results show that POs rely on communication with colleagues, and they work in a team-based approach to reach a consensus regarding difficult treatment decisions. Other researchers have stressed the importance of having experience in pediatric palliative care when working with children with cancer in order to prevent burnout and anxiety (27–29). In the present study, POs were purposely included based on having experience in informing and discussing treatment options in difficult situations, including enrolment in EPCTs. However, our study could not show that more experienced POs felt less emotionally involved. On the contrary, all POs declared facing various limitations around EPCTs regardless of their level of experience. A reason for this finding could be the few opportunities to acquire specific experience and knowledge due to the low number of EPCTs. Furthermore, several POs shared that this task affected them personally on an emotional level, particularly the least experienced PO. At the same time, the vast majority of POs stated that their emotional involvement did not negatively influence the informed consent process. In summary, and as previously described in Sweden (27), the role of being a messenger in a life-limiting situation appears to be the real challenge for POs regarding communication around EPCTs. These challenges go beyond their limitations in knowledge and experience.

In most cases, when an EPCT is introduced, there is an established patient-physician relationship. Crucial issues for the POs are to strive to maintain hope and assess whether enrolment in an EPCT is of benefit to the child or poses a risk of reduced quality of life. This means that the family’s wishes must be considered, and the information provided to the family has to be adapted according to the given situation. Swedish POs are accustomed to meeting and delivering information to the whole family together. Patient involvement is grounded in Swedish law, which is clearly outlined in the Patient Act, 2014:821 (30) as the right for children to be asked about healthcare decisions and to pay regard to their opinion in decision-making according to the United Nations Convention on the Rights of the Child, 2018:1197 (31). Shared decision-making (SDM) is a way to optimize patient participation, and in accordance with the literature a good relationship supports the SDM process (32, 33). Participation in an EPCT goes one step further, and patients must give consent according to the Review Ethical Act, 2003:460 (34); to be able to do so, patients need to understand what they consent to (9). Shared decision-making, a challenging process in standard healthcare (35), is further complicated by the informed consent process (22, 36) in the context of an EPCT, and made even more difficult when applied to children in the palliative stages. Yet, when applied appropriately, SDM can improve healthcare decisions and maintain the patient’s best interest (37, 38). The POs in this study wanted to involve the families as much as possible but sometimes found it hard to include them in all decisions. A challenge regarding SDM deals with the fact that, according to Swedish law (The Patient Act, 2014:821) (30), POs are fully accountable for all treatment-related decisions. These decisions and responsibilities can never be handed over to patients or parents. Furthermore, parents of children with cancer report that they are reluctant to talk to healthcare professionals about sensitive issues, such as their child’s prognosis (39, 40). One reason is fear of the answers they might receive, particularly in the presence of their child. This is likely related to the fact that parents, as well as older children, need a meeting place with medical professionals where they themselves can talk about the situation and the future and freely express their feelings of worry and grief. The strength of the study is the mixed method design and a national approach that considered the entire population of POs in Sweden regarding pediatric oncologists’ experiences with EPCTs. A limitation could be the lack of representation of other healthcare professionals. On the other hand, only the medical profession, in this study represented by POs, have the responsibility of referring, enrolling and/or treating children in EPCTs. The study results are based on a comparison between two groups of POs with different levels of experience within pediatric oncology. Another possibility would have been to divide the groups based on the number of ECPTs that each PO had been involved in. Further studies involving affected families and their experiences are needed to enhance the family’s involvement in communication around EPCTs and the SDM process.

## 5 Conclusion

The number of EPCTs involving children with cancer is consistently increasing in Sweden. In line with the literature on pediatric palliative care (13), POs who provide care for children eligible for EPCT inclusion express needs related to education and training in communication (40). Overall, POs shared their experiences of working in a field where one cannot simply rely on expertise and previous experience and where one is probably deeply emotionally involved. In line with other healthcare professionals the POs have articulated the need for supervision, training in communication, and participation in seminars, and stressed the importance of having the opportunity to discuss treatment options and ethical aspects around EPCTs with colleagues. The results can form the basis for a national educational initiative with a focus on the professional aspects of working with integrated pediatric palliative care in EPCTs, including medical, psychosocial, communication, and ethical issues.

## Data Availability

The raw data supporting the conclusions of this article will be made available by the authors, without undue reservation.
